# Cerebrovascular Reactivity Measurement Using Magnetic Resonance Imaging: A Systematic Review

**DOI:** 10.3389/fphys.2021.643468

**Published:** 2021-02-25

**Authors:** Emilie Sleight, Michael S. Stringer, Ian Marshall, Joanna M. Wardlaw, Michael J. Thrippleton

**Affiliations:** ^1^Centre for Clinical Brain Sciences, University of Edinburgh, Edinburgh, United Kingdom; ^2^UK Dementia Research Institute, Edinburgh, United Kingdom

**Keywords:** cerebrovascular reactivity, magnetic resonance imaging, blood oxygen-level dependent, arterial spin labelling MRI, Hypercapnia (CO(2)) inhalation, systematic review

## Abstract

Cerebrovascular reactivity (CVR) magnetic resonance imaging (MRI) probes cerebral haemodynamic changes in response to a vasodilatory stimulus. CVR closely relates to the health of the vasculature and is therefore a key parameter for studying cerebrovascular diseases such as stroke, small vessel disease and dementias. MRI allows *in vivo* measurement of CVR but several different methods have been presented in the literature, differing in pulse sequence, hardware requirements, stimulus and image processing technique. We systematically reviewed publications measuring CVR using MRI up to June 2020, identifying 235 relevant papers. We summarised the acquisition methods, experimental parameters, hardware and CVR quantification approaches used, clinical populations investigated, and corresponding summary CVR measures. CVR was investigated in many pathologies such as steno-occlusive diseases, dementia and small vessel disease and is generally lower in patients than in healthy controls. Blood oxygen level dependent (BOLD) acquisitions with fixed inspired CO_2_ gas or end-tidal CO_2_ forcing stimulus are the most commonly used methods. General linear modelling of the MRI signal with end-tidal CO_2_ as the regressor is the most frequently used method to compute CVR. Our survey of CVR measurement approaches and applications will help researchers to identify good practice and provide objective information to inform the development of future consensus recommendations.

## Introduction

Cerebrovascular reactivity (CVR) reflects the ability of the blood vessels to dilate in order to match tissue blood supply to increased demand and can be investigated by measuring the change in cerebral blood flow (CBF) or cerebral blood volume (CBV) that vasodilation induces. It is a valuable tool for assessing vascular health in pathologies, including steno-occlusive diseases (Mandell et al., 2008b), while more subtle CVR impairments have been found in Alzheimer's disease (Chen, 2018) and cerebral small vessel disease (Wardlaw et al., 2019). The measurement of CVR relies on three key elements: the vasodilatory stimulus, the signal acquisition and the processing method.

### Vasodilatory Stimulus

Vasodilation occurs naturally as a mechanism of CBF auto-regulation, but can also be triggered by exogenous stimuli inducing extracellular and intracellular acidosis. The resulting decrease in pH relaxes smooth muscle cells lining the arteries and arterioles, thereby increasing their diameter. Common stimuli include changes in arterial CO_2_ partial pressure (PaCO_2_) induced by voluntary modulations of the breathing pattern, including breath-holding, hyperventilation and paced breathing (Petersson and Glenny, 2014; Urback et al., 2017; Liu et al., 2019) or by inhalation of CO_2_-enriched gas (Fierstra et al., 2013; Liu et al., 2019). As PaCO_2_ cannot easily be measured *in vivo*, end-tidal CO_2_ (EtCO_2_), the most recent maximal exhaled CO_2_ partial pressure, is often used as a surrogate and can be measured by recording the CO_2_ level in the exhaled gas using a gas monitor. Several approaches exist to manipulate PaCO_2_: inhalation of gas with fixed CO_2_ concentration (e.g., CO_2_-enriched air or carbogen), rebreathing the exhaled gas, EtCO_2_ targeting manually or using a computer-controlled device (Fierstra et al., 2013). Vasodilation can be induced without modulating the composition of the inhaled gas or breathing pattern by injection of acetazolamide (ACZ), a carbonic anhydrase inhibitor that causes acidosis (Vagal et al., 2009).

### Signal Acquisition

Several imaging methods can assess haemodynamic changes induced by the vasodilatory stimulus. Positron emission tomography (PET), single-photon emission computed tomography (SPECT) (Ogasawara et al., 2003) and computed tomography (CT) (Marion and Gerrit, 1991) have all been used to measure CVR, but involve ionising radiation and have low temporal resolution. Transcranial Doppler ultrasound is a practical alternative, but has a limited field of view that allows blood velocity measurements only in parts of single large vessels, which do not necessarily reflect local changes in tissue blood supply (Purkayastha and Farzaneh, 2012; McDonnell et al., 2013). Magnetic resonance imaging (MRI) is a non-invasive, non-ionising technique which allows CVR mapping using contrasts related to CBF and/or CBV. Arterial spin-labelling (ASL) and phase-contrast (PC) MRI measure CBF in tissue and large vessels, respectively (Valdueza et al., 1997; Noth et al., 2008), while vascular space occupancy (VASO) MRI measures CBV (Donahue et al., 2009). Dynamic susceptibility contrast (DSC)-MRI measures both CBF and CBV (Taneja et al., 2019) by monitoring the *T*_2_ or *T*_2_^*^-weighted signal following intravenous injection of a gadolinium-based contrast agent. Blood Oxygen Level Dependent (BOLD) imaging, using a *T*_2_ or *T*_2_^*^-weighted sequence, can also measure CVR due to its sensitivity to a combination of CBF and CBV.

### Processing Method

The signal change due to the vasodilatory stimulus must be converted into a quantitative or semi-quantitative measurement of CVR using one of several methods. Pre-vs.-post-stimulus subtraction of the MRI signal relies on the computation of the absolute or relative signal difference before and after the stimulus has been applied (Donahue et al., 2013; Wu et al., 2017). Often, the pre- and post-values are calculated by taking the average of the MRI volumes acquired during each period respectively, discarding volumes that are acquired during the transition period. Linear regression is a method that investigates the linear relationship between the dependent variable (in this case the MRI signal or derived CBF) and independent variables (e.g., EtCO_2_, to reflect the vasodilatory stimulus; time, to model a linear signal drift) (Thrippleton et al., 2018; Liu et al., 2019), allowing the MRI time course to be modelled using multiple predictors simultaneously. Cross-correlation quantifies the similarity between two signals (e.g., the MRI signal and EtCO_2_) as a function of their relative time delay (Donahue et al., 2016) and has been used as a measure of CVR. Non-linear fitting involves modelling the MRI signal as a non-linear function (Ziyeh et al., 2005; Germuska et al., 2019). It requires some initial estimate of the CVR and other parameters such as CVR delay, and can be more challenging to implement than linear regression, but has the advantage that any models can be used to fit the MRI signal. Some models (e.g., calibrated fMRI models) also allow quantitative estimation of CVR and other parameters that can be of interest such as cerebral metabolic rate of oxygen (CMRO_2_). Frequency-based analysis includes transfer function (Duffin et al., 2015) and Fourier (Blockley et al., 2011) analyses. In both methods, the signals of interest (e.g., the MRI signal and EtCO_2_) are transformed into the frequency domain. The magnitude of the signal at the stimulus frequency is then defined as the CVR. Finally, the standard deviation of the MRI signal (Kannurpatti et al., 2014; Jahanian et al., 2017) can be computed as a metric of CBF change due to natural vasodilation and vasoconstriction.

### Aims of the Review

Since many combinations of the above stimuli, imaging methods and analysis techniques are possible, there are potentially many different ways to measure CVR *in-vivo*, resulting in a high degree of methodological diversity in the literature. Previous reviews described common CVR-MRI experiments (Fierstra et al., 2013; Pillai and Mikulis, 2015; Moreton et al., 2016; Urback et al., 2017; Liu et al., 2019) or CVR data analysis (Fisher et al., 2018). However, as far as we are aware, there are no systematic reviews detailing the breadth of CVR-MRI acquisition techniques, processing methods and applications that have been presented and used in the literature.

We conducted a systematic literature review of papers reporting the use of CVR-MRI techniques. We present an overview of the different aspects of the CVR-MRI experiment reported and applied in the literature, describing the most common methods and clinical research applications. We classified and systematically analysed reports of the MRI techniques, vasodilatory stimuli, data processing methods and study populations. Based on these findings we identified recent practises, trends, technical findings and evidence from clinical studies to inform future application and standardisation of CVR-MRI protocols.

## Materials and Methods

### Search Strategy

We systematically reviewed the EMBASE and MEDLINE databases from 1980, until June 2020 using Ovid. The search strategy combined terms relating to: “Cerebrovascular reactivity,” “MRI,” “BOLD,” “ASL,” “PC,” “hypercapnia,” “acetazolamide,” and “CO_2_.” We manually added relevant articles from the authors' libraries. The search was not constrained to English-language literature. Full details of the search strategy are provided as Supplementary Information.

### Eligibility Criteria

We included all studies that investigated changes in cerebral blood flow or cerebral blood volume using MRI due to vasodilation or vasoconstriction in humans. We excluded reviews, conference abstracts, editors' notes, and case reports (single-subject studies focussed on methodological aspects of CVR were included). We removed studies that did not investigate induced vasodilation in the brain or used another imaging modality (e.g., CT, PET) to measure CVR. Studies that measured the change in the BOLD signal in response to a functional task and hypercapnia but did not compute a CVR metric were also excluded.

### Data Extraction

One author (E.S.) screened the titles and abstracts of all potentially eligible publications to exclude duplicates and assess eligibility against the inclusion criteria before reading the full text of the remaining articles to determine eligibility. Eligibility and data extraction were discussed with other authors where queries around inclusion or exclusion, or data extraction arose.

We extracted population characteristics, including pathology, sample size, age, and gender. We recorded MRI acquisition parameters including magnetic field strength, type of pulse sequence and sequence parameters (e.g., TR, TE, spatial resolution, field-of-view). We recorded the type of vasodilatory stimulus, measurement of EtCO_2_ and/or end-tidal O_2_ (EtO_2_), stimulus paradigm and, where available, information on tolerability, number and reason for any excluded or failed scans. Finally, we extracted information on the pre-processing steps, delay correction/computation methods and CVR processing methods applied, reported grey and white matter CVR values in healthy volunteers and relevant findings.

## Results

### Search Results

**Figure 1:**
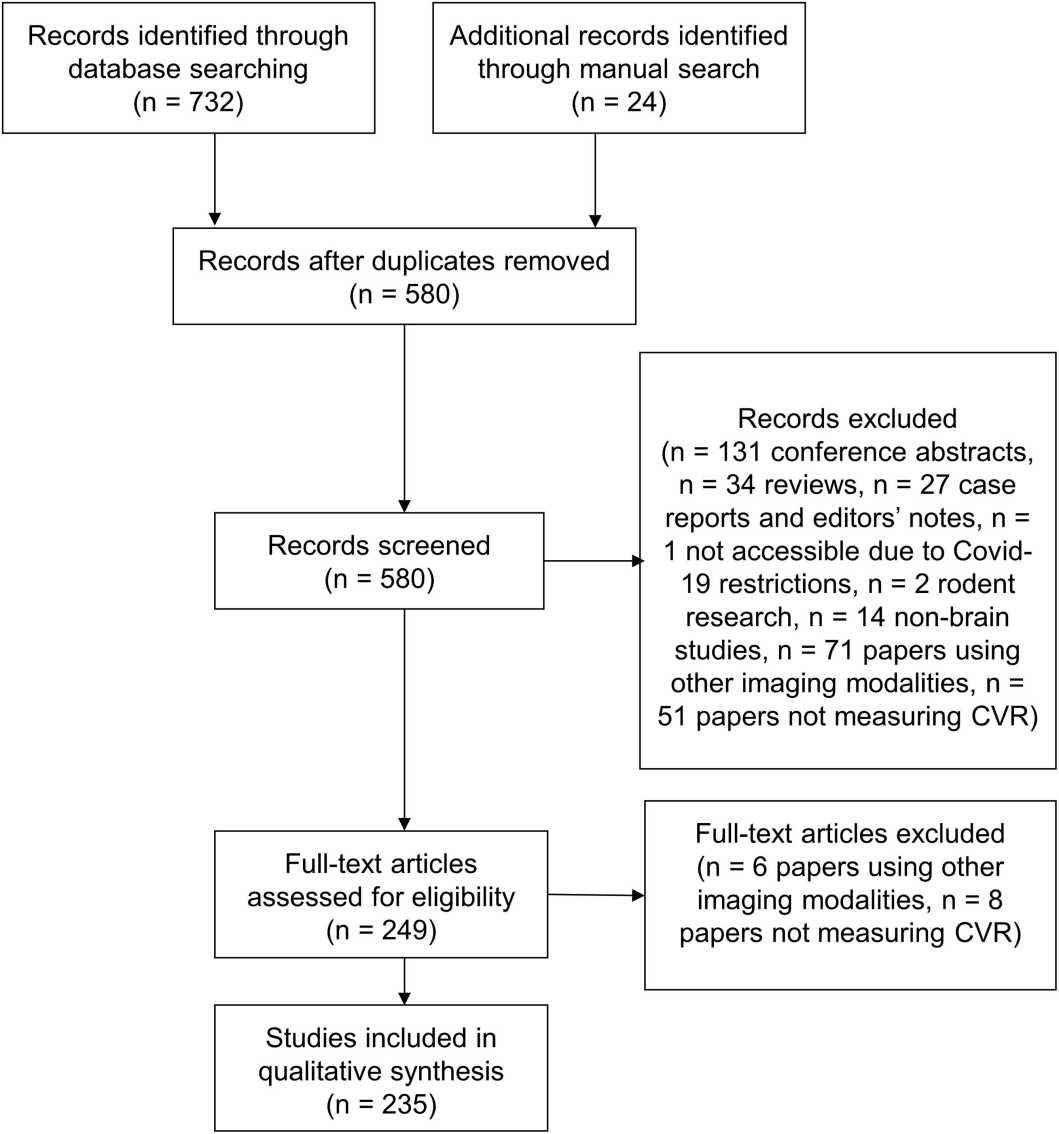
Flow diagram of the literature search.

We identified 732 articles, 176 of which were removed as duplicates (Figure 1). Of the remaining 556 papers, 317 were excluded on review of the title and abstract due to a lack of analysable data or insufficient detail [*n* = 192: conference abstracts (*n* = 131), reviews (*n* = 34), and case reports and notes to the editor (*n* = 27)], inaccessibility (*n* = 1), only reporting rodent studies (*n* = 2), using other modalities (e.g., PET, TCD, CT, SPECT) (*n* = 71) and not measuring CVR (*n* = 51). After full text review an additional 14 papers were removed because they used other imaging modalities to measure CVR (*n* = 6) or did not measure CVR (*n* = 8). Additionally, 24 articles were added from the authors' libraries. We included 235 papers in the review. Summary data extracted from each study is included in the Supplementary Material.

### Population Characteristics

The studies included 5,369 unique participants. 36 subjects were excluded before CVR due to contraindication to MRI (*n* = 6) or ACZ (*n* = 3), claustrophobia in the MRI scanner (*n* = 5), too large to fit in the MRI scanner (*n* = 1), anxiety during pre-testing of the stimulus (*n* = 1) and intolerance of the stimulus (*n* = 20). The remaining 5,333 unique participants who had a CVR scan comprised 2,394 patients and 2,939 healthy participants. All studies reported a sample size, with a mean sample size of 35 (median: 19, range: 1–536). Forty-five studies had fewer than 10 subjects whereas 9 included more than 100 subjects. Twelve papers did not report any age information, a further 18 papers reported only the age range. The mean age, computed as the mean of the mean or median ages, was 44.3 (1.4–92) years. The median gender distribution was 43% females and 57% males, excluding the 18 studies not reporting gender distribution.

The total number of scans including longitudinal scans was 7,437. The number of scans excluded from analyses was 518/7,437 (7%), not including scans that were selected from a database for being of good quality. Per study, the mean percentage of datasets excluded from analysis was 6% (range: 0–38%). Scans were excluded for one or more reasons: incomplete dataset (28/518, 5%), subject's discomfort (79/518, 15%), irregular breathing (3/518, 1%), non-compliance (38/518, 7%), technical issues (67/518, 13%), pre-processing issues (5/518, 1%), poor data quality (40/518, 8%), motion artefacts (183/518, 35%), outlier CVR values (13/518, 3%), non-CVR related (75/518, 14%, e.g., post-operative stroke, resolution of stenosis, hematoma, issue with therapeutic intervention) and no reasons reported (2/518, 0.3%).

Information on tolerability of the CVR experiment ranged from information regarding subject withdrawal to subjective rating of tolerability and was reported in 51/235 (22%) studies (1,162/5,333 unique subjects). Overall, the CVR experiment in these 51 studies was mostly described as well-tolerated. One article studied the tolerability of 434 CVR (294 subjects) scans acquired with EtCO_2_ targeting BOLD MRI and concluded that it was well-tolerated (Spano et al., 2013). Six studies reported subjective tolerability: the experiment was rated as tolerable to very tolerable with minimal discomfort on average in each study. Twenty-three studies detailed complaints of discomfort: 11 studies reported no complaints or adverse effects whereas 12 did. These 12 studies (618 subjects) reported 120 complaints transient to the CVR scan: respiratory symptoms due to gas inhalation such as breathing resistance and shortness of breath (*n* = 77), anxiety and/or claustrophobia (*n* = 16), dizziness and/or headache (*n* = 10), narrowness of head coil with gas apparatus (*n* = 4), tachycardia (*n* = 3), paraesthesia (*n* = 3), chest tightness (*n* = 1), conjunctive erythema (*n* = 1), tremor (*n* = 1), hand weakness (*n* = 1), nausea, confusion, and blurred vision (*n* = 1) and no details of the complaints (*n* = 2). No long-lasting symptoms were reported and no studies using acetazolamide injection detailed complaints or adverse effects. In 17 studies, 79 scans were defined as untolerable by the subject due to: anxiety (*n* = 21), claustrophobia (*n* = 16), discomfort related to gas apparatus in the scanner (*n* = 9), position in the head coil (*n* = 2) and no details (*n* = 31).

### Pathologies

Cerebral steno-occlusive diseases (e.g,. Moyamoya disease, carotid stenosis/occlusion) were the most commonly investigated diseases (72/235 studies, 31%), followed by dementia and cognitive impairments (9/235, 4%), normal ageing (8/235, 3%), small vessel disease (7/235, 3%), sport-related concussions (7/235, 3%), obstructive sleep apnoea (5/235, 2%), stroke (5/235, 2%; one of which also investigated CVR in steno-occlusive disease), traumatic brain injury (4/235, 2%), tumours (4/235, 2%), diabetes with or without hypertension (3/235, 1%), and miscellaneous (18/235, 8%). Of the 142 articles reporting CVR measurements in pathology (referred to in Table 1), 70 studies assessed CVR to investigate pathophysiology, 48 studies explored the technical feasibility of a methodology to detect CVR impairment, 13 studies investigated the effect of a therapeutic intervention on CVR (surgical intervention for steno-occlusive diseases such as revascularisation, candesartan therapy for diabetes, bariatric surgery for obese subjects, haemodialysis for end-stage renal disease, therapeutic continuous positive airway pressure for obstructive sleep apnoea), six studies investigated the progression of pathologies, and five studies looked at the effect of CVR on fMRI BOLD activation. Relative to healthy controls, CVR was lower in patients in most of the pathologies (Krainik et al., 2005; Donahue et al., 2009; da Costa et al., 2016; Hartkamp et al., 2018; Thrippleton et al., 2018; McKetton et al., 2019) and CVR delays were longer in steno-occlusive diseases, small vessel disease and dementia (Hartkamp et al., 2012; Duffin et al., 2015; Thrippleton et al., 2018; Atwi et al., 2019; Holmes et al., 2020).

**Table 1:** Pathologies in which CVR was investigated.

**Pathology**	**Number of studies**	**Number of patients/controls**	**Mean age of patients/controls**	**Findings**	**References**
Cerebral steno-occlusive diseases	72	1786/541	51.4/44.9	- Lower CVR than healthy controls (Hartkamp et al., 2017; Liu et al., 2017a; De Vis et al., 2018; Duffin et al., 2018; Venkatraghavan et al., 2018; Waddle et al., 2019) - Longer delays than healthy controls (Hartkamp et al., 2012; Duffin et al., 2015; Liu et al., 2017b; Waddle et al., 2019) - Increased CVR (Han et al., 2011a; Mandell et al., 2011; Watchmaker et al., 2019) and smaller delays (Watchmaker et al., 2019) after surgical intervention	Piepgras et al., 1994; Guckel et al., 1995; Ohnishi et al., 1996; Schreiber et al., 1998; Hamzei et al., 2003; Shiino et al., 2003; Griffiths et al., 2005; Ziyeh et al., 2005; Ma et al., 2007; Haller et al., 2008; Mandell et al., 2008b, 2011; Chang et al., 2009, 2013; Donahue et al., 2009, 2013, 2016; Goode et al., 2009, 2016; Calviere et al., 2010; Bokkers et al., 2011; Conklin et al., 2011; Han et al., 2011a,b; Kim et al., 2011; Uchihashi et al., 2011; Hartkamp et al., 2012, 2017, 2018, 2019; Mutch et al., 2012; Poublanc et al., 2013, 2015; Spano et al., 2013; Thomas B. et al., 2013; Donahue et al., 2014; Sam et al., 2014, 2015; Sobczyk et al., 2014, 2015, 2016; Bouvier et al., 2015; De Vis et al., 2015b, 2018; Duffin et al., 2015, 2017, 2018; Faraco et al., 2015; Noguchi et al., 2015; Siero et al., 2015a; Herrera et al., 2016; Strother et al., 2016; van Niftrik et al., 2016; Dlamini et al., 2017, 2018; Federau et al., 2017; Fisher et al., 2017; Hu et al., 2017; Ladner et al., 2017; Liu et al., 2017a,b; Para et al., 2017; Wu et al., 2017; Fierstra et al., 2018b; Rosen et al., 2018; Sebok et al., 2018; Venkatraghavan et al., 2018; Hauser et al., 2019; Taneja et al., 2019; Waddle et al., 2019; Watchmaker et al., 2019; Papassin et al., 2020
Dementia and cognitive impairment	9	770/125	60.5/68.1	- Lower CVR than healthy controls (Cantin et al., 2011; Yezhuvath et al., 2012) - Longer delays than healthy controls (Holmes et al., 2020) - Higher CVR deficit vs. healthy controls associated with higher leukoaraiosis (Yezhuvath et al., 2012) and hypertension (Haight et al., 2015) - Lower CVR in the bilateral frontal cortices of Alzheimer's patients compared to patients with vascular dementia (Gao et al., 2013)	Cantin et al., 2011; Yezhuvath et al., 2012; Gao et al., 2013; Haight et al., 2015; Richiardi et al., 2015; Suri et al., 2015; Lajoie et al., 2017; McKetton et al., 2019; Holmes et al., 2020
Normal ageing	8	NA/374	Range: [20, 90]	- Lower CVR at older ages (Riecker et al., 2003; Liu et al., 2013; De Vis et al., 2015a; Bhogal et al., 2016; Leoni et al., 2017; Catchlove et al., 2018; Miller et al., 2019) - Greater WM CVR and shorter delay with increasing age (Thomas et al., 2014)	Riecker et al., 2003; Liu et al., 2013; Thomas et al., 2014; De Vis et al., 2015a; Bhogal et al., 2016; Leoni et al., 2017; Catchlove et al., 2018; Miller et al., 2019
Small vessel disease	7	272/54	67.4/45.7	- Lower CVR with increased WMH burden (Liem et al., 2009; Blair et al., 2020) and compared to healthy controls (Liem et al., 2009; Tchistiakova et al., 2015; Thrippleton et al., 2018; Atwi et al., 2019) - Longer delays than healthy controls (Sam et al., 2016a; Thrippleton et al., 2018; Atwi et al., 2019) - Reduced WM CVR associated with higher WMH volumes, basal ganglia PVS and higher venous pulsatility and lower foramen magnum CSF stroke volume (Blair et al., 2020) - Lower baseline CVR associated with progression of WMHs but not microbleeds or lacunar infarcts (Liem et al., 2009) - Lower CVR associated with increased number of vascular risk factors such as hypertension, diabetes, hypercholesterolemia (Tchistiakova et al., 2015), lower fractional anisotropy, lower CBF and CBV and higher mean diffusivity (Sam et al., 2016b) - Lower CVR and longer delays in NAWM that progressed into WMH (Sam et al., 2016a)	Liem et al., 2009; Tchistiakova et al., 2015; Sam et al., 2016a,b; Thrippleton et al., 2018; Atwi et al., 2019; Blair et al., 2020
Sport-related concussions	7	113/128	18.6/21.2	- Lower CVR in the default mode network at mid-season and 1 month post-season compared to pre-season baseline. Decrease in CBF occurred only 1 month after season (Champagne et al., 2019c) - Longitudinal reduction in CVR during season compared to pre-season baseline was associated with prolonged accumulation to high magnitude acceleration events (Svaldi et al., 2020) - Predominant increase in CVR compared to baseline during the recovery phase but remains mostly altered despite clinical recovery (Mutch et al., 2016b) - Higher CVR in clinically recovered patients with history of concussions than in athletes without (Mutch et al., 2018; Champagne et al., 2019b)	Mutch et al., 2016b, 2018; Champagne et al., 2019b,c; Champagne et al., 2020a; Coverdale et al., 2020; Svaldi et al., 2020
Obstructive sleep apnoea	5	125/55	50.6/44.5	- Greater CVR than in healthy controls measured using ASL with BH, BOLD with BH (Wu et al., 2020) and BOLD with EtCO_2_ targeting (Ryan et al., 2018). - ASL response to fixed CO_2_ enriched air reduced in patients with OSA compared to healthy controls, whereas BOLD response to fixed CO_2_ enriched air or BH did not show group differences (Ponsaing et al., 2018).	Buterbaugh et al., 2015; Ponsaing et al., 2018; Ryan et al., 2018; Thiel et al., 2019; Wu et al., 2020
Stroke	5	135/102	58.7/51.0	- Lower CVR in impaired tissue and compared to healthy controls (Krainik et al., 2005; Zhao et al., 2009; Geranmayeh et al., 2015) - Higher CVR with increasing distance from lesion (Taneja et al., 2019) - Reduced CVR associated with peri-infarct T2 hyperintensities, greater infarct volume and worse outcomes (Zhao et al., 2009) - Reduced CVR in the motor areas controlling the upper airway musculature compared to healthy controls (Buterbaugh et al., 2015). - No change in CVR upon CPAP withdrawal (Thiel et al., 2019)	Krainik et al., 2005; Zhao et al., 2009; Geranmayeh et al., 2015; Raut et al., 2016
Traumatic brain injury	4	90/77	32.2/31.8	- Lower CVR than healthy controls in one study (Amyot et al., 2018) - No difference in CVR between patients and healthy controls in one study (Champagne et al., 2020b) - Lower GM CVR with more concussion symptoms (da Costa et al., 2016)	da Costa et al., 2016; Mutch et al., 2016a; Amyot et al., 2018; Champagne et al., 2020b
Gliomas	4	50/12	43.9/not reported	- Lower CVR on ipsilateral side for low and high grade gliomas	Hsu et al., 2004; Pillai and Zaca, 2012; Zaca et al., 2014; Fierstra et al., 2018a
Diabetes	3	103/32	67.5/61.8	- Lower CVR in diabetic hypertensive patients than in only hypertensive patients (Kario et al., 2005; Tchistiakova et al., 2014) - Higher CVR in bilateral pre-frontal lobe in one study (Zhou X.-H. et al., 2015) - Increased CVR after candesartan therapy (Kario et al., 2005)	Kario et al., 2005; Tchistiakova et al., 2014; Zhou X.-H. et al., 2015
Pathologies investigated in two studies:					
- Cardia index and coronary artery disease (Anazodo et al., 2016; Jefferson et al., 2017)					
- Sickle cell disease (Leung et al., 2016a; Kosinski et al., 2017)					
- Multiple sclerosis (Metzger et al., 2018; Sivakolundu et al., 2019)					
- Obesity (Frosch et al., 2017; Tucker et al., 2020)					
- Brain arteriovenous malformation and cerebral proliferative angiopathy (Fierstra et al., 2011a,b)					
- Parkinson's disease (Al-Bachari et al., 2014; Pelizzari et al., 2019)					
Pathologies investigated in one study:					
- End-stage renal disease (Zheng et al., 2016)					
- Bipolar disorder (Urback et al., 2019)					
- Late-life depression (Abi Zeid Daou et al., 2017)					
- Late-onset epilepsy (Hanby et al., 2015)					
- HIV (Callen et al., 2020)					
- Aneurysmal subarachnoid haemorrhage (Da Costa et al., 2014)					
- MELAS (Rodan et al., 2015)					

*“Mean age” was computed by taking the average across studies of the reported mean/median age of the patients. GM, grey matter; WM, white matter; WMH, white matter hyperintensity; NAWM, normal-appearing white matter; CPAP, continuous positive airway pressure; MELAS, Mitochondrial encephalomyopathy, lactic acidosis and stroke-like episodes; HIV, Human immunodefiency viruses; CSF, cerebral spinal fluid; NA, not applicable; OSA, obstructive sleep apnoea; BH, breath-hold*.

### MRI Technique

**Figure 2:**
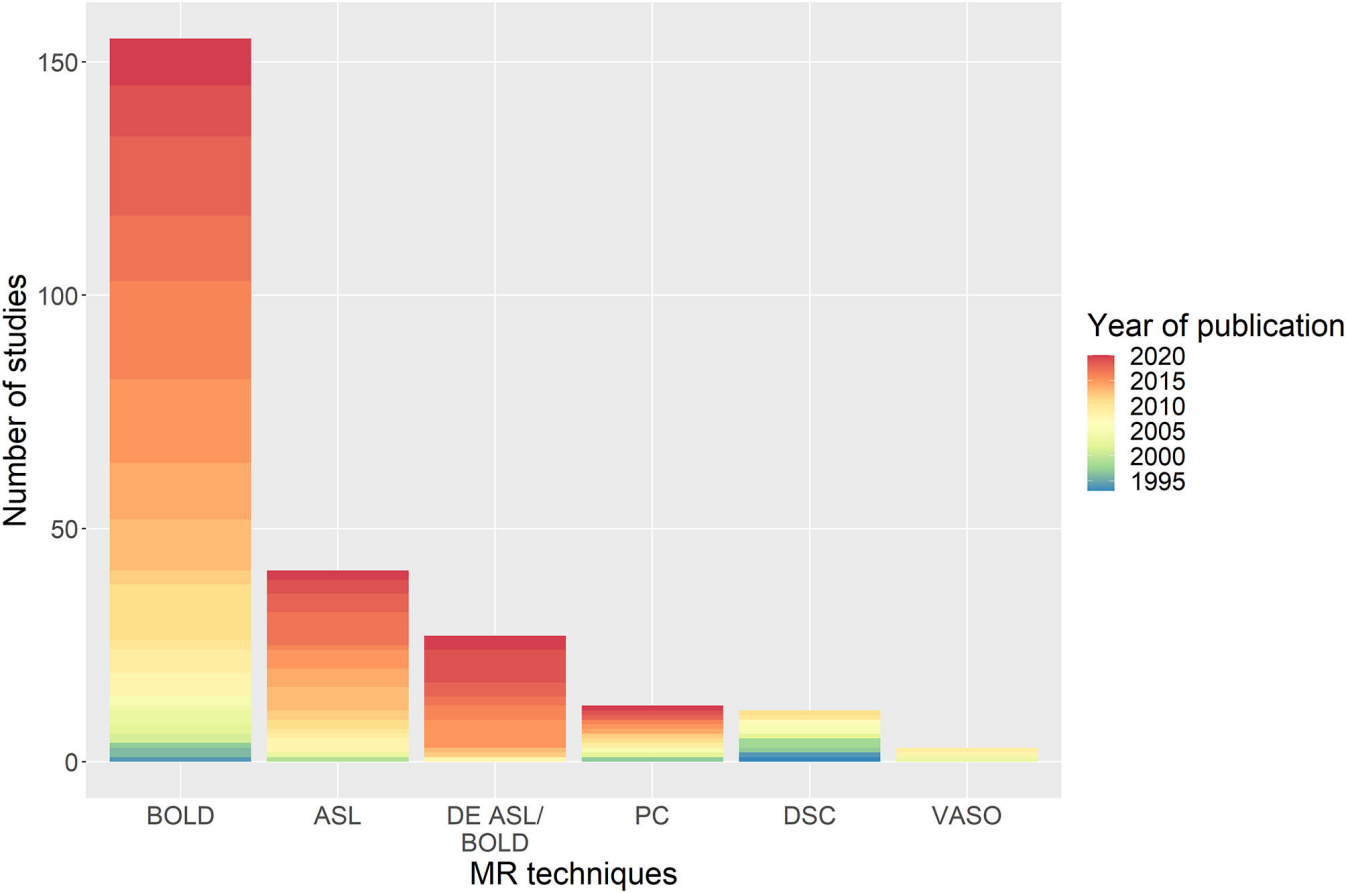
Distribution of the MRI sequences used in studies with the associated year of publication of the paper. BOLD, blood-oxygen-level-dependent; ASL, arterial spin-labelling; DE, dual-echo; PC, phase-contrast; DSC, dynamic susceptibility contrast; VASO, vascular space occupancy.

The number of CVR-MRI studies that were conducted at 3 T is 178/235 (74%), with the remainder acquired at: 1.5 T (47/235, 20%), 7 T (9/235, 4 %), 2 T (2/235, 1%) and a combination of 1.5 and 3 T (3/235, 1%). Studies used one or more MRI techniques to acquire CVR data (Figure 2): BOLD (155/235, 66%), ASL (41/235, 17%), dual-echo providing simultaneous ASL and BOLD data (27/235, 11%), PC (12/235, 5%), DSC (11/235, 5%), and VASO (3/235, 1%). In recent publications, BOLD, ASL and dual-echo ASL/BOLD are the most common MRI techniques. Summary MRI parameters for the BOLD gradient-echo echo-planar imaging (GE-EPI), pulsed continuous ASL (pCASL) and dual-echo ASL/BOLD GE-EPI techniques at 3 T are given in Table 2.

**Table 2:** Median values and ranges of MRI parameters at 3 T found in the literature.

**MRI technique**	**Number of studies**	**TR (ms)**	**TE (ms)**	**Flip angle (^**°**^)**	**Spatial resolution (mm)**	**3D FOV (mm)**	**PLD (ms)**	**Label duration (ms)**	**GRAPPA/SENSE (number of studies)**	**SMS (number of studies)**
BOLD GE-EPI	118	2,000 [66.4, 3,000]	30 [20, 50]	85 [40, 90]	3.44 × 3.44 × 4 [1.72 × 1.72 × 2, 4 × 4 × 8]	225 × 220 × 126 [192 × 150 × 18, 256 × 256 × 195]	-	-	2 [1.8, 3] (14)	3 [2, 6] (2)
pCASL GE-EPI	16	4,000 [3,000, 4,500]	14 [10, 20]	90 [90, 90]	3 × 3 × 6 [3 × 3 × 4, 7 × 7 × 7]	240 × 240 × 119 [220 × 220 × 54, 240 × 240 × 135]	1,525 [900, 2,000]	1,650 [1,500, 1,650]	2.5 [2, 2.5] (8)	(0)
Dual-echo pCASL/BOLD GE-EPI	22	4,000 [3,000, 4,500]	TE_1_ = 10 [8.4, 16]. TE_2_ = 30 [25, 47]	90 [90, 90]	3.44 × 3.44 × 5 [3 × 3 × 3.9, 4.5 × 4.5 × 7]	240 × 240 × 116.1 [192 × 192 × 87, 256 × 256 × 132]	1,220 [600, 1,868]	1,650 [1,200, 2,000]	2 [2, 2.3] (10)	(0)

*PLD, post-labelling delay; TR, repetition time; TE, echo time; FOV, field-of-view; GE, gradient-echo; EPI, echo-planar imaging; SMS, simultaneous multi-slice*.

Three studies (*n* = 18) found BOLD-derived CVR values were lower at lower magnetic field strengths (Driver et al., 2010; Triantafyllou et al., 2011; Peng et al., 2020), two of which (*n* = 9) reported a linear relationship between BOLD-derived CVR and the field strength (Driver et al., 2010; Triantafyllou et al., 2011). In one study (*n* = 16), ASL-derived CVR did not differ at different field strengths (Noth et al., 2006). One study (*n* = 8) reported longer post-labelling delay results in lower baseline CBF and ASL-CVR measurements (Inoue et al., 2014). Use of EPI with parallel imaging compared to spiral imaging, reduced signal loss due to susceptibility-induced magnetic field gradients in BOLD-CVR measurements without affecting sensitivity, which was defined as the CVR t-statistic (*n* = 5) (Winter et al., 2009). Furthermore, one study (*n* = 5) showed that using simultaneous multi-slice acceleration of factor 2 and 3, can reduce scan duration by at least a half compared to conventional EPI while maintaining the CVR sensitivity (Ravi et al., 2016a). Compared to single-echo ASL or BOLD EPI, a multi-echo (four echoes) EPI acquisition followed by T2^*^ fitting of the signal decay had higher inter-scan repeatability of breath-hold CVR analysed across voxels, CVR sensitivity and test-retest reliability analysed using the intra-class correlation coefficient (*n* = 14) (Cohen and Wang, 2019).

### Vasodilatory Stimulus

**Figure 3:**
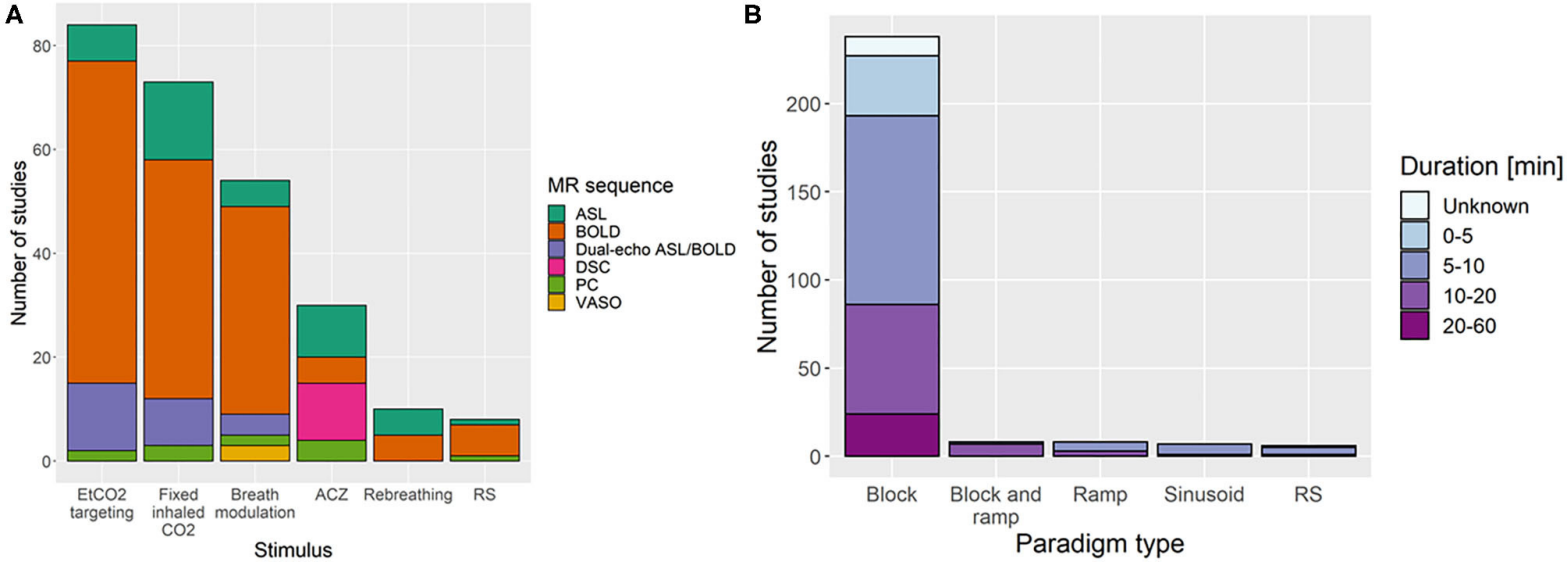
Distribution of the **(A)** stimuli with the associated MRI sequence and **(B)** paradigm types with associated total duration of the CVR experiment. In **(A)**, the “breath modulation” stimulus includes breath-holding, paced breathing, and hyperventilation stimuli. ACZ, acetazolamide injection; RS, resting-state; BOLD, blood oxygen-level dependent; ASL, arterial spin-labelling; PC, phase contrast; DSC, dynamic susceptibility contrast; VASO, vascular space occupancy.

To induce vasodilation, several stimuli were employed in the literature (Figure 3A): EtCO_2_ targeting manually or using a computer-controlled device such as RespirAct (Thornhill Research, Toronto, Canada) (81/235 studies, 34%), fixed inspired gas administration (69/235, 29%), breathing modulations (52/235, 22%), ACZ injection with median dose of 1 g (29/235, 12%), rebreathing (10/235, 4%), resting-state haemodynamic fluctuations (8/235, 3%) and not reported (1/235, 0.4%). Three different fixed inspired gases were identified: CO_2_-enriched (67%), O_2_-enriched (i.e. hyperoxia, 10%), and CO_2_- and O_2_-enriched air (i.e. carbogen, 23%). In some studies, these gas compositions were alternated during the same paradigm with or without intermittent normal air periods using the fixed inspired gas, EtCO_2_ targeting methods: alternating hypercapnia and hyperoxia (15/235, 6%), alternating CO_2_-enriched air and carbogen (1/235, 0.4%). For fixed inspired CO_2_ paradigms, the median percentage of inhaled CO_2_ was 5% (range: 2–10%). While the combination of MRI sequence and stimulus generally varied across studies DSC-MRI was used only with ACZ injection. Block design paradigms were most common (212/235 studies, 90%) with a median stimulus plateau duration of 1 min. The median total experiment duration was 9 min (Figure 3B). One study did not specify the type of paradigm, and 12 further studies did not report the duration of the CVR experiment.

Removing studies that used ACZ stimulus, 160/207 studies measured EtCO_2_ (77%) of which 21 did not report the targeted or achieved EtCO_2_ variation (14%), 80 studies also measured EtO_2_ (39%). The median EtCO_2_ change induced by the stimulus was 9 mmHg (range: 2.2–28 mmHg). Seventy-five studies reported mean baseline EtCO_2_ at rest (47%), with a median value of 39 mmHg (range: 31.2–43.4 mmHg). 21% of the studies that used EtCO_2_ targeting controlled the baseline EtCO_2_ (40 mmHg for all studies) instead of using the individual EtCO_2_ value when breathing normal air.

One study (*n* = 4) found BOLD response to EtCO_2_ is 60 times higher than to EtO_2_, but demonstrated that during hypercapnic CVR-BOLD experiments, EtO_2_ should be controlled if the change in EtCO_2_ is small compared to the change in EtO_2_ (Prisman et al., 2008). One study (*n* = 9) demonstrated that carbogen should not be used with BOLD or ASL to measure CVR due to a lack of correlation between both MRI techniques as opposed to CVR measurements using CO_2_-enriched air with BOLD or ASL (Hare et al., 2013). Another study (*n* = 20) found that, for a gas challenge, an effect of at least 2 mmHg EtCO_2_ change is required to detect haemodynamic impairment using BOLD at 3 T (De Vis et al., 2018). RS-BOLD was found to give CVR results that were associated with fixed-inspired CO_2_ BOLD (*n* = 48, Liu et al., 2017a) and RespirAct BOLD (*n* = 13, Golestani et al., 2016) measurements. One study (*n* = 8) reported differences in response amplitude and onset time depending on whether BH was performed before and after expiration (Leoni et al., 2008). For BOLD-BH, one study (*n* = 6) demonstrated that the fraction activation volume saturated for breath-hold durations of 20 s and above; thus recommended using breath-hold durations of 20 s to give sufficient sensitivity to BOLD signal changes to detect impaired CVR (Liu et al., 2002).

### CVR Data Processing Methods

**Figure 4:**
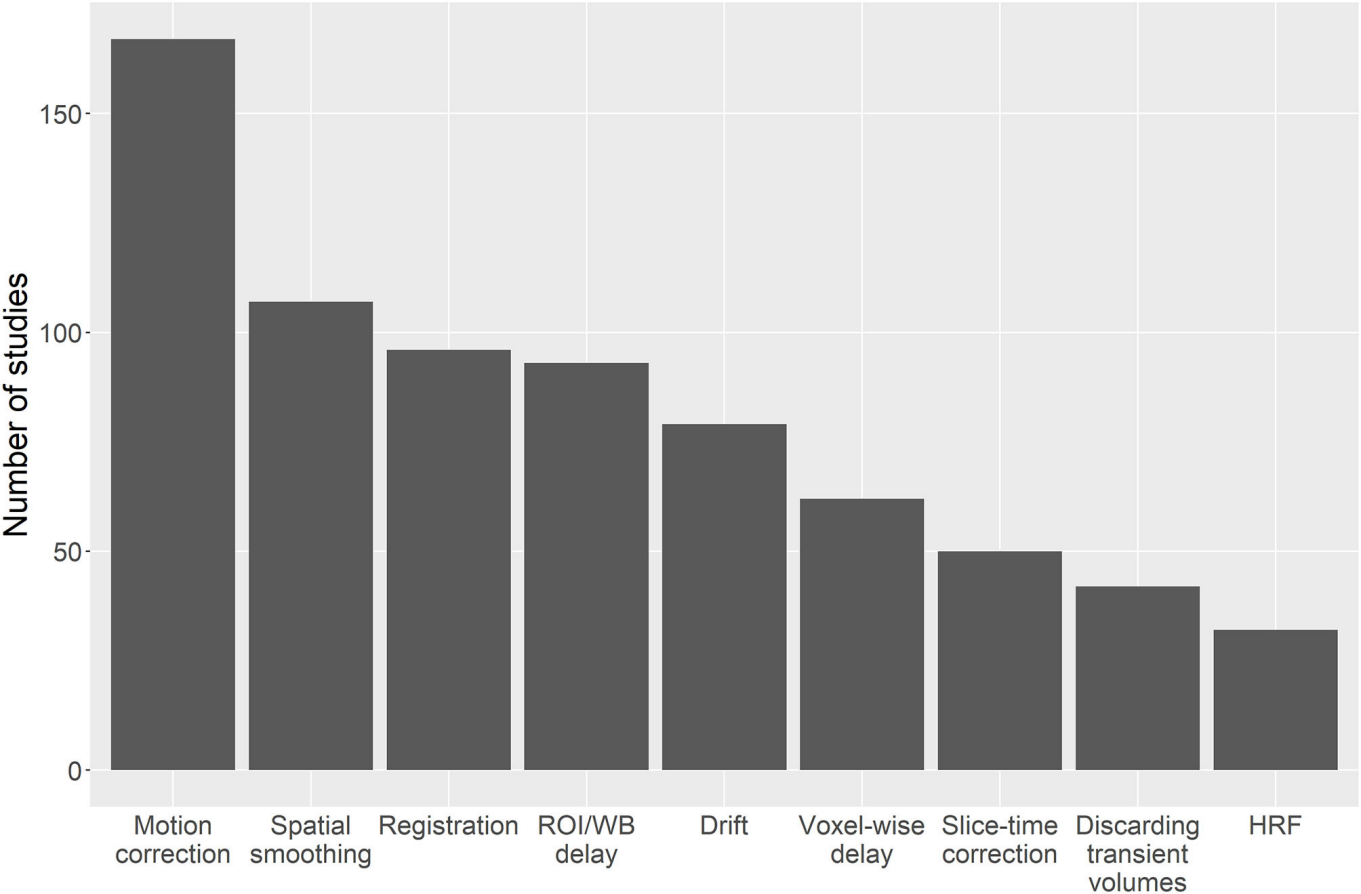
Bar chart showing the number of studies that apply different pre-processing steps. ROI, region of interest; WB, whole brain; HRF, haemodynamic response function.

Common pre-processing steps that were reported (Figure 4) were sequence-dependent and included motion correction (167/235 studies, 71%), spatial smoothing (107/235, 46%), registration of functional volumes to MNI or subject space (96/235, 41%), region-of-interest or whole brain delay correction (93/235, 40%), drift removal/modelling (79/235, 34%), voxel-wise delay correction (62/235, 26%), and discarding transient MRI volumes to consider only those where steady-state signal was reached (42/235, 18%). Only 3% of papers corrected for sampling line delay. Slice-time correction was used in 51 of 180 BOLD/DE-BOLD studies. Eroding the edges of the regions of interest can reduce vascular contamination of CVR due to larger responses to CO_2_ in blood vessels than in tissues (Thrippleton et al., 2018). T1 correction was recommended for CVR-ASL data involving the use of carbogen or other hyperoxic gas because of changes in the longitudinal relaxation time during hyperoxia (*n* = 24, Siero et al., 2015b). The most common software packages used for pre-processing and/or CVR analysis were Statistical Parametric Mapping (SPM, 89/235 studies, 38%), in-house Matlab (The Mathworks, Natick, MA, United States) software (90/235, 38%), FMRIB Software Library (FSL, 65/235, 28%), and Analysis of Functional NeuroImages (AFNI, 54, 23%) (some studies used more than one package in combination). Only one in-house Matlab script (for pre-processing BOLD and EtCO_2_ data) reported to be publicly available (Lu et al., 2014).

**Figure 5:**
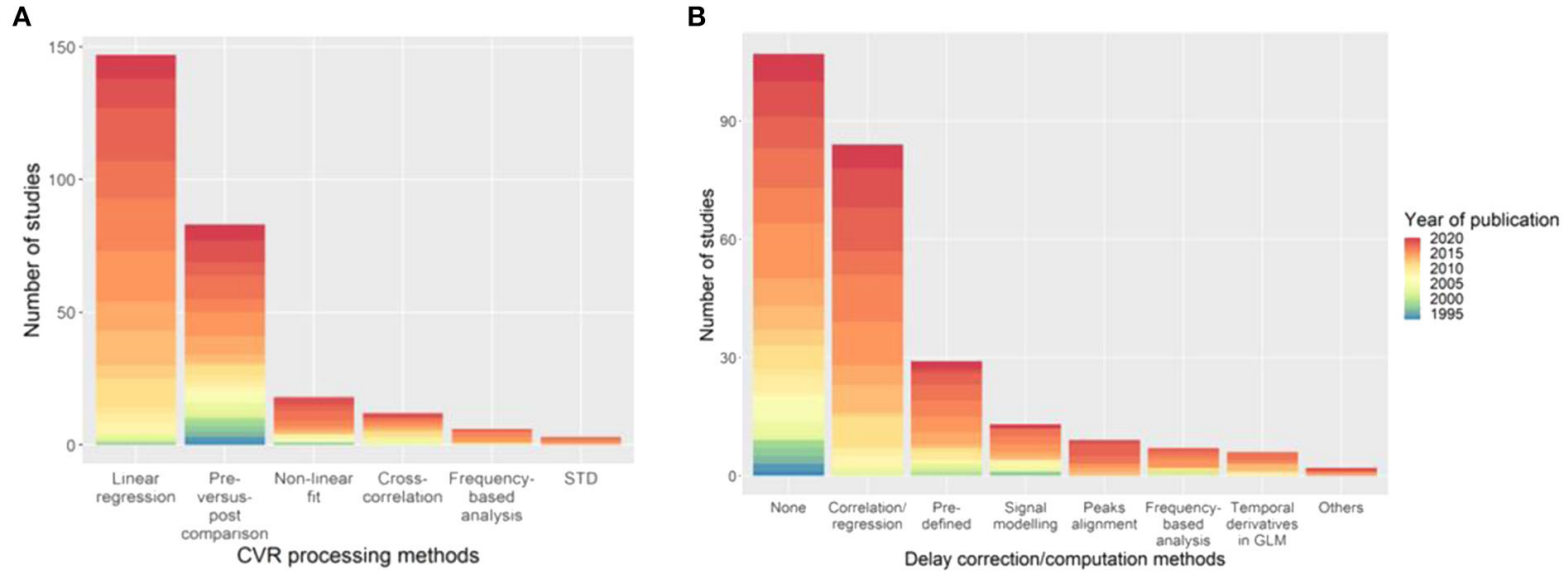
Distribution of the **(A)** CVR processing and **(B)** delay computation methods with the associated year of publication of the paper. The category “Others” in **(B)** includes deconvolution to find the HRF between the EtCO_2_ and the MRI signal, and GLM with two (“fast” and “slow”) regressors. STD, standard deviation of MRI signal; HRF, haemodynamic response function; GLM, general linear model.

Of the six classes of CVR calculation methods identified, linear regression is the most common method overall (149/235 studies, 63%) and in recent publications. However, several newer methods are under development including frequency-based analysis (Duffin et al., 2015). The main reference signal used to compute linear regression or cross-correlation is the EtCO_2_ (89/235 studies, 38%). An HRF was incorporated in the MRI signal model in 14% of the studies (32/235), with the single or double gamma function being the most common choice (22/235 studies, 9%). A relatively new method to find an appropriate regressor is RIPTiDe (Regressor Interpolation at Progressive Time Delays), which derives the reference signal from the MRI data by iteratively applying principal component analysis on aligned MRI time courses until convergence of the regressor (Tong et al., 2011; Donahue et al., 2016). Twenty one studies did not clearly describe the CVR processing method, of which two included no information, these were excluded from the summary of CVR processing method (Figure 5A).

Dynamic aspects of CVR (e.g., lung-to-brain delay, response time) were computed in 128/235 studies (54%) using different methods (Figure 5B), however some studies used different MRI techniques and multiple associated delay processing methods. Fourteen of the delay computation methods were not clearly described and 8 of which could not be included in Figure 5B. Cross-correlation- and linear regression-based methods can be used to compute CVR delay by determining the time shift that gives the best correlation between the BOLD signal and a reference signal (e.g., EtCO_2_). The most common delay computation methods are cross-correlation- or, equivalently, linear regression-based approaches (84/128 studies, 66%) and pre-defined delay, e.g., from literature, voxel examination, (29/128, 23%). The delay between two signals can be found using linear regression or cross-correlation by determining the time shift giving the best correlation between these two signals. As with CVR computation, delay computation is an evolving area and new methods are arising including obtaining the delay directly from the HRF between the BOLD signal and EtCO_2_ (Atwi et al., 2019). One study corrected the hypercapnic delay for delay due to the vasculature (i.e., the delay it takes for the blood and CO_2_ to travel from the lungs to the brain tissues) by using the BOLD delay from a hyperoxia challenge as a surrogate of vasculature delay and assuming no vasodilation due to hyperoxia (Champagne et al., 2019a). This correction can distinguish between delay due to vasculature and delay due to vasodilation.

CVR values in whole brain, grey and white matter of healthy volunteers are summarised in Table 3. The associated processing methods were linear regression (72/104, 69%), pre-vs.-post stimulus value comparison (17/104 values, 16%), non-linear signal modelling (13/104, 13%) and frequency-based analysis (2/104, 2%). CVR in grey matter was higher than CVR in white matter. Moreover, measuring white matter CVR using ASL is not common, probably due to the fact that ASL suffers from low contrast-to-noise ratio (CNR) (Liu et al., 2019).

**Table 3:** Mean/median CVR values at 3 T in healthy volunteers as a function of the age range (in the square brackets are the minimum and maximum values and in the round brackets is the number of values and number of subjects used to compute the mean).

**Age range**	**Whole brain**		**Grey matter**		**White matter**	
	**%BOLD signal change/mmHg**	**%CBF/mmHg**	**%BOLD signal change/mmHg**	**%CBF/mmHg**	**%BOLD signal change/mmHg**	**%CBF/mmHg**
<30	0.19/0.18 [0.14, 0.24] (6 studies, 94 subjects)	4.5 (1 study, 16 subjects)	0.35/0.26 [0.05, 1.80] (17 studies, 294 subjects)	3.69/3.6 [1.9, 6.6] (7 studies, 124 subjects)	0.12/0.12 [0.03, 0.29] (12 studies, 236 subjects)	1.1 (1 study, 18 subjects)
30–50	0.22/0.22 [0.11, 0.28] (15 studies, 169 subjects)	4.64 (1 study, 16 subjects)	0.26/0.26 [0.14, 0.44] (10 studies, 127 subjects)	3.60/3.28 [2.40, 5.11] (3 studies, 45 subjects)	0.12/0.13 [0.08, 0.18] (8 studies, 101 subjects)	-
>50	0.21/0.21 [0.15, 0.31] (6 studies, 120 subjects)	3.58/3.4 [2.2, 5.30] (4 studies, 279 subjects)	0.36/0.36 [0.13, 1.30] (6 studies, 124 subjects)	2.12/2.13 [2.10, 2.15] (2 studies, 21 subjects)	0.13/0.12 [0.05, 0.33] (5 studies, 117 subjects)	-

*The associated processing methods were linear regression, pre-vs.-post stimulus value comparison, non-linear signal modelling and frequency-based analysis*.

### Repeatability, Reproducibility, and Accuracy of CVR Measurements

CVR values determined using MRI were generally found to be similar or well-correlated with those obtained using other imaging modalities such as PET, SPECT or TCD (Table 4: 10 studies, 193 subjects). Within- and between-day repeatability of MRI was studied mostly in healthy participants and in some stroke and steno-occlusive patients (Table 5: 14 studies, 191 subjects). The reported coefficients of variation show that CVR measurements are less repeatable between- than within-days (Kassner et al., 2010; Dengel et al., 2017) and less repeatable in white matter than in grey matter due to lower CNR in white matter (Kassner et al., 2010; Thrippleton et al., 2018).

**Table 4:** Comparison of CVR values measured using MRI vs. alternative imaging modalities.

**References**	**MRI technique**	**Comparator**	**Population**	**Results**
Ziyeh et al. (2005)	BOLD; fixed inhaled CO_2_	TCD; fixed inhaled CO_2_	20 SOD	Pearson CC for signal change in MCA territory: *r* = 0.71
Fierstra et al. (2018b)	BOLD; EtCO_2_ targeting	PET; ACZ	16 SOD, 10 HC	- Pearson CC for CVR difference unaffected vs. affected hemisphere: *r*^2^ = 0.47 - Pearson CC for CVR difference unaffected vs. affected hemisphere in MCA territory: *r*^2^ = 0.61
Herrera et al. (2016)	BOLD; BH	TCD; BH	15 SOD, 7 HC	- Cohen's kappa coefficient for the visual classification of normal or impaired CVR in the ipsilateral lentiform nucleus between the two methods: Overall, κ = 0.54; Controls, *κ* = −0.69; Patients, *κ* = 0.43
Shiino et al. (2003)	BOLD; BH	SPECT; ACZ	10 SOD, 17 HC	- Linear correlation between mean whole brain %BOLD change with mean whole brain change in CBF from SPECT: *r* = 0.70
Hauser et al. (2019)	BOLD; BH	PET; ACZ	20 SOD	- Spearman CC for maps: *r* = 0.90 - Pearson CC for relative signal change in vascular territories relative to cerebellum: *r* = 0.71
Heijtel et al. (2014)	ASL; fixed inhaled CO_2_	PET; fixed inhaled CO_2_	16 HC	- Pearson CC for GM CBF: *r*^2^ = 0.30 for baseline, *r*^2^ = 0.12 for hypercapnia - GM CVR [%/mmHg]: 2.82 for ASL vs. 2.50 for PET - Inter-modality RI: 22.9% for baseline, 30.3% for hypercapnia
Uchihashi et al. (2011)	ASL; ACZ	SPECT; ACZ	20 SOD	- Spearman rank CC of mean relative CVR in frontal and temporal lobes: *r* = 0.88 - Accuracy: mean difference in CVR of frontal and temporal lobes: 1.3%
Ma et al. (2007) and Kim et al. (2011)	DSC; ACZ	SPECT; ACZ	17 (Kim et al., 2011) and 12 (Ma et al., 2007) SOD	- Wilcoxon signed rank test on percent change in mean relative CBF in GM MCA territory (Kim et al., 2011): - normal side: 0.76–0.18 - lesion side: 0.38–0.67 - Association between detection of CVR impairment with SPECT and reduced DSC-CVR (Ma et al., 2007)
Grandin et al. (2005)	DSC; ACZ	PET; ACZ	13 HC	- Inter-modality correlation (coefficient of determination) in individual subjects: *r*^2^ between 0.70 and 0.84 - Mean difference in CBF and CBV: 8.2 ml/min/100g and 2.09 ml/100g at rest, 5.7 ml/min/100g and 2,45 ml/100g after ACZ

*TCD, transcranial Doppler ultrasound; BH, breath-hold; BOLD, blood oxygen level-dependent; ACZ, acetazolamide; SPECT, single photon emission computed tomography; CC, correlation coefficient; CV, coefficient of variation; RI, repeatability index; GM, grey matter; DSC, dynamic susceptibility contrast; MCA, middle carotid artery; ICC, intraclass CC; HC, healthy controls; SOD, steno-occlusive disease; SD, standard deviation*.

**Table 5:** Findings of repeatability of CVR estimates.

**References**	**MRI technique**	**Repeatability**	**Population**	**Results**
Thrippleton et al. (2018)	BOLD; fixed inhaled CO_2_	Within-day repeatability	15 HC	- Inter-session CV of GM CVR: 7.9–15.4% for a 3 min and 11.7–70.2% for a 1 min challenge - Inter-session CV of WM CVR: 16.1–24.4% for a 3 min and 27.5–141.0% for a 1 min challenge
Sobczyk et al. (2016), Leung et al. (2016b) and Dengel et al. (2017)	BOLD; EtCO_2_ targeting	Within- (Leung et al., 2016b; Dengel et al., 2017) and between-day repeatability (Leung et al., 2016b; Sobczyk et al., 2016; Dengel et al., 2017) and within-day consecutive intra-scan repeatability (Dengel et al., 2017)	15 (Sobczyk et al., 2016),11 (Dengel et al., 2017), and 10 (Leung et al., 2016b) HC	- Within-day intra-scan ICC of GM CVR (Dengel et al., 2017): 0.84 - Within-day intra-scan CV of GM CVR (Dengel et al., 2017): 5.70% - Within-day inter-scan ICC CVR in GM: 0.78 (Dengel et al., 2017), 0.86 (Leung et al., 2016b); WM: 0.90 (Leung et al., 2016b) - Within-day inter-scan CV of GM CVR (Dengel et al., 2017): 6.62% - Between-day ICC CVR in GM: 0.69 (Dengel et al., 2017), 0.78 (Leung et al., 2016b); WM: 0.72 (Leung et al., 2016b) - Between-day CV CVR in GM: 7.87% (Dengel et al., 2017), 7.3% (Sobczyk et al., 2016); WM: 10.3% (Sobczyk et al., 2016)
Bright and Murphy (2013), Dlamini et al. (2018), and Peng et al. (2020)	BOLD; BH	Within-day (Dlamini et al., 2018; Peng et al., 2020), between-day (Peng et al., 2020), inter-regressors (Bright and Murphy, 2013) and intra-subject (Bright and Murphy, 2013) repeatability	20 SOD (Dlamini et al., 2018). 9 (Peng et al., 2020), 12 (Bright and Murphy, 2013) HC	- Within-day ICC of whole brain CVR: 0.7 (Dlamini et al., 2018), >0.4 (Peng et al., 2020) - Within-day CV of positive GM CVR: 9.1% (Dlamini et al., 2018), <33% (Peng et al., 2020) - Within-day CV of negative GM CVR (Dlamini et al., 2018): 22.5% - Inter-regressor ICC of GM CVR (Bright and Murphy, 2013): <0.4 for ramp regressor and 0.82 for EtCO_2_ regressor - Intra-subject ICC of GM CVR (Bright and Murphy, 2013): 1.03% for EtCO_2_ regressor
Sousa et al. (2014)	BOLD; paced deep breathing	Test-retest and inter-subject repeatability	9 HC	- Inter-subject CV of GM CVR: 20% - Intra-subject CV of GM CVR: 8% - Intra-subject ICC of GM CVR: 1.04
Kassner et al. (2010)	BOLD; EtCO_2_ forcing	Within, between-day and inter-subject repeatability	19 HC	- ICC of GM CVR: 0.92 within-day, 0.81 between-day - ICC of WM CVR: 0.88 within-day, 0.66 between-day - CV of GM CVR: 4.2% within-day, 6.8% between-day, 20% inter-subject - CV of WM CVR: 6.3% within-day, 9.9% between-day, 21.8% inter-subject
Liu et al. (2017a) and Taneja et al. (2019)	Resting-state BOLD	Within-day (Liu et al., 2017a) and between-day (Taneja et al., 2019) repeatability	6 stroke (Taneja et al., 2019), 10 HC (Liu et al., 2017a)	- Within-day ICC (Liu et al., 2017a): 0.91 - Between-day correlation of lesion CVR (Taneja et al., 2019): *r*^2^ = 0.91 - Between-day correlation of contralateral-to-lesion CVR (Taneja et al., 2019): *r*^2^ = 0.79
Heijtel et al. (2014)	ASL; fixed inhaled CO_2_	Within- and between-day repeatability	16 HC	- Within-day RI: 18.2%; Between-day RI: 25.1% for baseline CBF, 24.8% for hypercapnia CBF - CV of inter-subject variability: 12.9% for baseline CBF, 15.6% for hypercapnia CBF
Merola et al. (2018)	Dual-echo ASL/BOLD; fixed inhaled CO_2_	Within- and between-day repeatability	26 HC	- Correlation with coefficient of determination of GM CVR variability: 0.57 for within-day inter-scan, 0.41 for within-day inter-session, 0.02 for between-day - CV of inter-subject variability of GM CVR: 17.5% for within-day, 25% for between-day - CV of intra-subject variability for GM CVR: 9.5% for within-day inter-scan, 12.5% for within-day inter-session, 17.5% between-day
Grandin et al. (2005)	DSC; ACZ	Inter-scan repeatability	13 HC	- Repeatability using the SD of the mean difference in CBF between scans: 22.4% - Repeatability using the SD of the mean difference in CBV between scans: 18.2%

*BH, breath-hold; BOLD, blood oxygen level-dependent; ACZ, acetazolamide; CC, correlation coefficient; CV, coefficient of variation; GM, grey matter; DSC, dynamic susceptibility contrast; ICC, intraclass CC; HC, healthy controls; SOD, steno-occlusive disease; SD, standard deviation; RI, repeatability index*.

CVR-MRI measurements were also compared between MRI techniques. CVR-BOLD and CVR-ASL were well-correlated using fixed CO_2_ concentration (*n* = 127) (Hare et al., 2013; Donahue et al., 2014; Zhou Y. et al., 2015) and computer-controlled EtCO_2_ using RespirAct (*n* = 13) (Zande et al., 2005). One study found no correlation between CVR-BOLD and CVR-ASL using carbogen (*n* = 9) (Hare et al., 2013). Using acetazolamide CVR-DSC correlated well with CVR-BOLD (*n* = 16) (Wu et al., 2017), but there was a lack of agreement between CVR-DSC and PC-MRI (*n* = 8) (Spilt et al., 2002).

### The Relationship Between BOLD Response and PaCO2

The healthy BOLD signal response to CO_2_ was found to be sigmoidal in two studies (*n* = 18) (Tancredi and Hoge, 2013; Bhogal et al., 2014). The sigmoid model of the BOLD response to CO_2_ was used in a further three studies (*n* = 65) (Bhogal et al., 2015, 2016; De Vis et al., 2018). In four studies, vasodilatory resistance to CO_2_ was modelled using the BOLD response (Sobczyk et al., 2014; Duffin et al., 2017, 2018; McKetton et al., 2019). The relationship between resistance and CO_2_ was assumed sigmoidal due to the limited ability of the blood vessels to constrict and dilate (*n* = 141). One study (*n* = 32) suggested that steal phenomenon associated with some pathologies could alter the sigmoid relationship between CO_2_ and vasodilatory resistance (Sobczyk et al., 2014).

### Potential Confounders of CVR Analysis

When analysing CVR measurements, baseline MRI signal or EtCO_2_ values (Bhogal et al., 2016) can lead to misinterpretation of CVR data (Mandell et al., 2008a; Blockley et al., 2011). Higher baseline EtCO_2_ was associated with lower CVR (*n* = 291) (Halani et al., 2015; van Niftrik et al., 2018; Hou et al., 2019). Baseline CBF and CBV were lower with age (*n* = 81) (Petrella et al., 1998; Leung et al., 2016a; Leoni et al., 2017), by contrast one study suggested age-related differences in baseline CBF may result from differences in baseline EtCO_2_ (*n* = 46) (De Vis et al., 2015a).

Negative CVR clusters correspond to MRI responses anti-correlated to the stimulus. In some cases this might simply reflect long CVR delays if they are not appropriately modelled. Negative CVR could also reflect the steal phenomenon, where tissues with high CVR “steal” blood flow from other regions due to flow redistribution (Shiino et al., 2003; Mandell et al., 2008a; Han et al., 2011a,b; Sobczyk et al., 2014; Poublanc et al., 2015; Fisher et al., 2017; Para et al., 2017; McKetton et al., 2018; Venkatraghavan et al., 2018; Hartkamp et al., 2019). However, they usually appear in the deep white matter (Mandell et al., 2008a), near and in the ventricles (Blockley et al., 2011). Therefore, others have suggested that they may result from low CNR in the white matter tissues leading to spurious CVR values (Blockley et al., 2011), or from reduction in cerebrospinal fluid (CSF) partial volume due to vasodilation (Thomas B. P. et al., 2013; Bright et al., 2014; Ravi et al., 2016b). The latter effect can be diminished by shortening TE (Ravi et al., 2016b).

### CVR Definition and Units

CVR was defined differently across studies and was reported in several units: relative change in BOLD signal divided by absolute change in EtCO_2_ with %/mmHg units (110/235, 47%), relative change in CBF divided by absolute change in EtCO_2_ with %/mmHg units (36/235, 15%), relative change in BOLD signal with % units (50/235, 21%), relative change in BOLD signal divided by relative change in total haemoglobin concentration ([Hb]) with %/[Hb] units (1/235, 0.4%), relative change in BOLD signal divided by breath-by-breath O_2_-CO_2_ exchange ratio with % units (1/235, 0.4%), relative change in BOLD signal divided by relative change in EtCO_2_ with % units (1/235, 0.4%), relative change in BOLD signal during one period of breath-hold with %/s units (1/235, 0.4%), relative change in CBF with units % (27/235, 11%), relative change in CBF during one period of breath-hold with %/s units (1/235, 0.4%), absolute change in CBF with ml.100 g^−1^.min^−1^ units (5/235, 2%), absolute change in CBF divided by the change in EtCO_2_ with ml.100 g^−1^.min^−1^mmHg^−1^ units (2/235, 1%), absolute change in CBF divided by mean arterial pressure divided by change in EtCO_2_ with ml/min/mmHg^2^ (1/235, 0.4%), mean arterial pressure divided by change in CBF with mmHg.ml^−1^.min.100 g units (1/235, 0.4%), relative change in CBV with % units (*n* = 13), absolute change in CBV with ml.100 g^−1^ units (1/235, 0.4%), absolute change in BOLD signal divided by change in EtCO_2_ a.u./mmHg (2/235, 1%), relative change in T2^*^ with % units (1/235, 0.4%), absolute change in T2^*^ divided by change in EtCO_2_ with ms/mmHg units (1/235, 0.4%), absolute change in R2^*^ divided by change in EtCO_2_ with s^−1^/mmHg (1/235, 0.4%). A further nine CVR definitions had no units because CVR was defined as the correlation coefficient between two time courses (7/235, 3%) and two were defined as the absolute change in BOLD signal divided by the standard deviation of the baseline BOLD signal (1/235, 0.4%) or by the absolute change in mean cerebellum BOLD signal (1/235, 0.4%). One article described different resistance sigmoid parameters associated with CVR such as resting reserve or amplitude, i.e., extend of vascular resistance from resting EtCO_2_ state to maximum vasodilation and extend of vascular resistance from maximum vasoconstriction to maximum vasodilation, respectively. Both resting reserve and amplitude are resistance parameters in mmHg/nL/s.

## Discussion

We identified 235 papers using MRI to measure CVR including 5,333 subjects, which covered several different acquisition and analysis methods. Stimuli, paradigm and duration, sequences used for acquisition and processing methods varied considerably. We found several papers, which investigated specific aspects of the CVR-MRI experiment such as processing methods or reproducibility of CVR measurement, but sample sizes were often low, and validation studies remain limited. Reporting was also inconsistent.

### Reporting Standards

Most papers included sufficient detail on the acquisition of CVR data (222/235, 94%). Only 22% of studies (51/235) reported CVR tolerability, less than half of which (23/235, 10%) reported presence or absence of discomfort complaints which may affect suitability for some patient populations. Processing (214/235, 91%) including delay computation methods (114/128, 89%) were well-reported, though only 54% (128/235) accounted for delay.

### Clinical Populations

CVR was measured in several pathologies including steno-occlusive diseases, stroke, small vessel disease, brain injuries, and dementia. Patients generally had lower CVR compared to healthy participants (Krainik et al., 2005; Donahue et al., 2009; da Costa et al., 2016; Hartkamp et al., 2018; Thrippleton et al., 2018; McKetton et al., 2019), though in obstructive sleep apnoea findings were mixed. Delays were longer in steno-occlusive, small vessel disease and dementia patients than in healthy controls, but were not reported in other pathologies. CVR metrics have been associated with cerebrovascular dysfunction, disease severity, and response to interventions (including revascularisation surgery for steno-occlusive diseases). CVR is therefore a promising biomarker of haemodynamic impairment and changes with broad applicability.

### Acquisition

Most CVR studies used a 3 T scanner (178/235, 74%) and 2D GE-EPI BOLD sequence (118/235, 50%) for acquisition. While several different sequences can measure CVR, all have limitations. BOLD signal results from a complex interaction between CBF, CBV, haemoglobin concentration, oxygen extraction fraction, cerebral metabolic rate of oxygen consumption and arterial O_2_ partial pressure (Liu et al., 2019). Changes in any of these parameters can alter the BOLD signal; however, there is evidence that CBV and CBF change together during hypercapnia (Chen and Pike, 2010; Hoge, 2012) and that CVR-BOLD is well-correlated with CVR-ASL (Mandell et al., 2008b; Hare et al., 2013; Zhou Y. et al., 2015). Cerebral metabolic rate of oxygen consumption might change during hypercapnia; however it is thought that these changes are small for low levels of CO_2_ stimulus (Hoge, 2012). ASL allows direct measurement of CBF and is also widely used (41/235, 17%), but suffers from low CNR (Liu et al., 2019); differences in labelling duration and efficiency, and bolus arrival time can also potentially affect CVR estimation. Calibrated fMRI (9/235, 4%) using dual-echo BOLD/ASL allows simultaneous quantification of CVR and cerebral metabolism parameters (e.g., rate of oxygen consumption and oxygen extraction fraction) (Germuska et al., 2016, 2019; Merola et al., 2017, 2018). However, calibrated fMRI models depend on the initialisation values of model parameters, model assumptions such as the oxygen metabolism not being altered during hypercapnia and hyperoxia stimuli (Germuska and Wise, 2019), and are more complex to implement. PC-MRI (12/235, 5%) measures CVR at the large-vessel level and generally provides limited spatial coverage; although 4D phase-contrast flow imaging (Miller et al., 2019; Morgan et al., 2020) is developing rapidly, the long scan duration currently limits applicability for measuring CVR in patients. Several different paradigms were used, which varied in duration and number of repetitions. EtCO_2_ targeting (81/235, 34%) and fixed CO_2_-inhalation (69/235, 29%) are the most widely used vasodilatory stimuli with a block paradigm (212/235, 90%) with a median paradigm duration of 9 min. Fixed CO_2_-inhalation is easier to set up than EtCO_2_ targeting but the change in EtCO_2_ associated with a specific inspired CO_2_ concentration may vary between subjects. EtCO_2_ targeting allows precise control over the EtCO_2_ and paradigm but requires expensive, specialist equipment. 75% of respiratory challenge studies (160/207) measured ETCO_2_. However, in patients with lung diseases, using EtCO_2_ is not a direct surrogate for PaCO2 (Petersson and Glenny, 2014).

### Processing Methods

CVR was mainly computed using linear regression (149/235, 63%). Few studies described why a particular processing method or regressor was used, and comparisons between different methods are lacking (Bright et al., 2017). 40% of the studies (93/235) calculated a whole brain or single region-of-interest delay that was applied to all voxels. While this method may be relatively robust against noise, delay is known to vary between and within tissue types (Thrippleton et al., 2018; Atwi et al., 2019). However, only 26% of studies (62/235) accounted for voxel-wise delays. An HRF between the stimulus and MRI signal was used in only 14% of the studies (32/235). This might be because the CVR HRF is unknown and may vary between stimuli, paradigms and pathologies (Poublanc et al., 2015; Sam et al., 2016a). Assuming a non-delta-function HRF allowed delay and steady-state CVR to be investigated in parallel (Poublanc et al., 2015; Donahue et al., 2016), but can be more complex to implement and computationally demanding.

### Validation

CVR measurements and detection of CVR impairment using MRI and other imaging modalities [e.g., BOLD-CVR to TCD-CVR (Ziyeh et al., 2005), BOLD-CVR to SPECT-CVR (Shiino et al., 2003), DSC-CVR to PET-CVR (Grandin et al., 2005)] were well-correlated, validating the CVR-MRI experiment. Furthermore, biological validation such as results from studies comparing CVR in patients with steno-occlusive diseases and healthy controls, also supports use of CVR as a biomarker (Ziyeh et al., 2005; Bokkers et al., 2011; Uchihashi et al., 2011; Thomas B. et al., 2013; De Vis et al., 2015b). Preclinical CVR imaging is also a fast-growing field which has been applied in preclinical models of stroke, cancer and Alzheimer's disease (Wells et al., 2015; Lake et al., 2016; Gonçalves et al., 2017). Preclinical CVR studies predominantly follow similar approaches to human studies but involve additional considerations such as the effect of anaesthetic agents on resting CBF (Stringer et al., 2021). Isolated vessel preparations (Seitz et al., 2004; Joutel et al., 2010), laser speckle imaging (Lynch et al., 2020), and multiphoton microscopy (Joo et al., 2017; Kisler et al., 2017) can also assess CVR preclinically and may help improve understanding of how impaired vasoreactivity develops and further direct validation of CVR-MRI as a clinical biomarker of cerebrovascular health (Stringer et al., 2021). CVR measurements using MRI techniques showed lower repeatability between-days than within-days (Dengel et al., 2017; Merola et al., 2018). CVR measurements were also less repeatable in white matter than in grey matter due to a lower CNR (Kassner et al., 2010; Thrippleton et al., 2018). Studies with higher sample sizes and investigating reproducibility in different pathologies would be helpful to further validate the CVR-MRI experiment.

### Definition and Interpretation of CVR

The definition and units of CVR vary across studies depending on choice of sequence, stimulus, paradigm and analysis methods. However, CVR is most commonly reported as the relative change in BOLD signal (110/235, 47%) or CBF (36/235, 15%) per unit change in EtCO_2_ as %/mmHg.

Several aspects influence CVR values beyond the vasodilatory capacity of vessels, which must be considered in interpreting the results. The CVR steal phenomenon has been proposed as a systemic mechanism governing the cerebrovascular system by prioritising blood supply to specific regions and potentially leading to local deficits elsewhere. Low or negative CVR values may also result from low CNR or blood vessel dilation near the ventricles shrinking the CSF space and artificially decreasing the BOLD signal due to differences in CSF and blood signal intensities (Thomas B. P. et al., 2013; Bright et al., 2014; Ravi et al., 2016b). Excluding voxels that contain CSF or using a shorter TE (e.g., 21 ms for a TR of 1,500 ms at 3T) can reduce negative artefacts in CVR data (Ravi et al., 2016b). Other physiological factors can affect the MR signal, including resting CBF and oxygen extraction fraction. Finally, as blood vessels have a limited vasodilation capacity, the linearity of the MRI response to the vasodilatory stimulus has a restricted range. Indeed, the shape of the MRI response to the stimulus and baseline parameters including resting CBF and EtCO_2_ can influence CVR values (Bhogal et al., 2014, 2016; van Niftrik et al., 2018; Hou et al., 2019). Despite some gaps in current knowledge, CVR has a proven validity and utility in several diseases as described above.

### Definition and Interpretation of CVR Delay

Delay in the MRI response to a stimulus can lead to inaccurate CVR values if it is not accounted for, and could give further information on vascular health. Voxel-wise or ROI delay should be favoured as opposed to whole brain delay to better account for differences in tissue response and distance from blood vessels. Artificially high or low delay values can be obtained when the noise level is high, i.e., low CNR. Definitions of delay were inconsistent in distinguishing between lung-to-brain delay and duration of the vasodilation process (Thomas et al., 2014). For example, one study computed the lung-to-brain delay, assuming instantaneous MRI response, as the shift in the EtCO_2_ that gives the lowest residual when regressed against the MRI time course: the delay in grey and white matter were approximately 15 and 35 s, respectively (Thomas et al., 2014). Another study computed the response time using a mono-exponential fit of the MRI signal: they found response time constants between a few seconds in grey matter up to 100 s in white matter (Poublanc et al., 2015).

### Implications for Future Research

#### Harmonisation of the CVR-MRI Experiment

Variability in the implementation of CVR experiments, including the choice of sequence and MRI parameters, such as TR and TE for BOLD MRI and post-labelling delay for ASL MRI (Inoue et al., 2014), causes heterogeneity in the CVR values, making it challenging to interpret findings across studies and conduct meta-analyses. CVR measurements are highly dependent on MRI sequence (e.g., BOLD, ASL, PC, and DSC), since each measures a different quantity as an estimate or surrogate of CBF, which are not directly comparable (Zhou Y. et al., 2015).

Harmonisation of acquisition and processing methods would allow more uniform definitions of CVR, delay and HRF, enhancing inter-study comparability, although specific techniques may be better suited to some pathologies and patient groups. Such efforts may also find consensus on the optimal paradigm duration to ensure that CO_2_ reaches the region of interest and the MRI response reaches the steady state. As many groups have developed in-house software to process CVR data, making these publicly available, as a step towards development of validated, community-driven open-source software, would also promote reproducibility and harmonisation.

While little consensus currently exists, our review reveals evidence of convergence in some aspects of the CVR-MRI experiment: the use of BOLD at 3T with a block paradigm for the acquisition and definition of CVR as the relative change in BOLD signal per unit change in mmHg (%/mmHg). Early attempts to establish a framework for reaching consensus have recently been initiated (Bright et al., 2017). Further work is needed to reach consensus regarding signal processing and CO_2_ delivery methods. CVR is also highly dependent on the image analysis methods used, including the erosion of regions of interest to avoid signal contamination from neighbouring tissues, or region of interest vs. voxel-wise analysis.

#### Considerations for Future Studies

Detailed reporting of methods and results is essential for interpretation and inter-study comparison of CVR data. Future publications should give sufficient detail to allow processing to be reproduced and, where possible, authors should release their software in version controlled open-source repositories. Results should preferably be reported in relative signal units to allow inter-study comparisons. Accurate recording and reporting of tolerability and reasons for excluding CVR scans is also important to facilitate clinical translation.

Non-linearity due to the limited vasodilation capacity of the blood vessels, is a consideration when interpreting CVR values. In this case, CVR reflects both the maximum response as well as the sensitivity to CO_2_. Research is needed to identify the aspects of the CVR response (e.g., maximum response, MRI response vs. EtCO_2_ slope) that are most sensitive and specific in key pathologies. Accounting for voxel-wise lung-to-brain delay would allow direct comparison of the BOLD signal and EtCO_2_ and should improve the accuracy of CVR values. Drift in the MRI signal can be significant and should be controlled for during signal processing (Liu et al., 2019).

Finally, there are age-related changes in CVR values: CVR is lower with increasing age in grey and white matter (Thomas et al., 2014; Leung et al., 2016c; Leoni et al., 2017). Statistical analyses should account for such key covariates, which requires larger sample sizes or matched study design. CVR is also associated with vascular risk factors including hypertension, diabetes, hypercholesterolemia and smoking (Haight et al., 2015; Tchistiakova et al., 2015; Sam et al., 2016b; Blair et al., 2020).

### Strengths and Weaknesses

This review included foreign language papers (5/236), though one such paper was inaccessible. Most but not all of the required information was obtained during the data extraction. This might have added a bias to the results of this review: for example, description of the CVR processing and delay computation methods were not clear in 9% and 11% of the studies, respectively. Furthermore, the sample size of many studies was low (mean sample size: 35, 45/235 studies ≤ 10 participants), particularly in studies investigating repeatability and reproducibility of CVR values (mean: 16). This review was also restricted to human studies; therefore it does not provide a detailed description of preclinical CVR methods, although the main processing techniques are similar.

## Conclusion

To our knowledge, this is the first systematic review to summarise and describe the diverse acquisition and analysis techniques used to measure CVR using MRI, and their applications in health and disease. While CVR-MRI is a relatively new and evolving technique we identified applications in several clinical populations including steno-occlusive and small vessel disease, highlighting the value of CVR measurements in medical research. However, acquisition techniques, analysis methods and definitions of CVR all varied substantially. Future work should target consensus recommendations to facilitate more reliable and harmonised CVR measurement for use in clinical research and trials of new therapies.

## Data Availability Statement

The original contributions presented in the study are included in the article/Supplementary Material, further inquiries can be directed to the corresponding author.

## Author Contributions

ES performed the search, analysed the data, and prepared the manuscript. MT, JW, MS, and IM contributed to the work by discussing the eligibility and data extraction of some papers and by reviewing the manuscript. All authors contributed to the article and approved the submitted version.

## Conflict of Interest

The authors declare that the research was conducted in the absence of any commercial or financial relationships that could be construed as a potential conflict of interest.
