# Corrigendum: Cerebrovascular reactivity measurement using magnetic resonance imaging: A systematic review

**DOI:** 10.3389/fphys.2022.1105285

**Published:** 2022-12-09

**Authors:** Emilie Sleight, Michael S. Stringer, Ian Marshall, Joanna M. Wardlaw, Michael J. Thrippleton

**Affiliations:** ^1^ Centre for Clinical Brain Sciences, University of Edinburgh, Edinburgh, United Kingdom; ^2^ UK Dementia Research Institute, Edinburgh, United Kingdom

**Keywords:** cerebrovascular reactivity, magnetic resonance imaging, blood oxygen-level dependent, arterial spin labelling MRI, hypercapnia (CO(2)) inhalation, systematic review

In the published article, there was an error in Figure 5 as published: the wrong Figure 5A was inserted. The corrected Figure 5 and its caption appear below.

**FIGURE 5:**
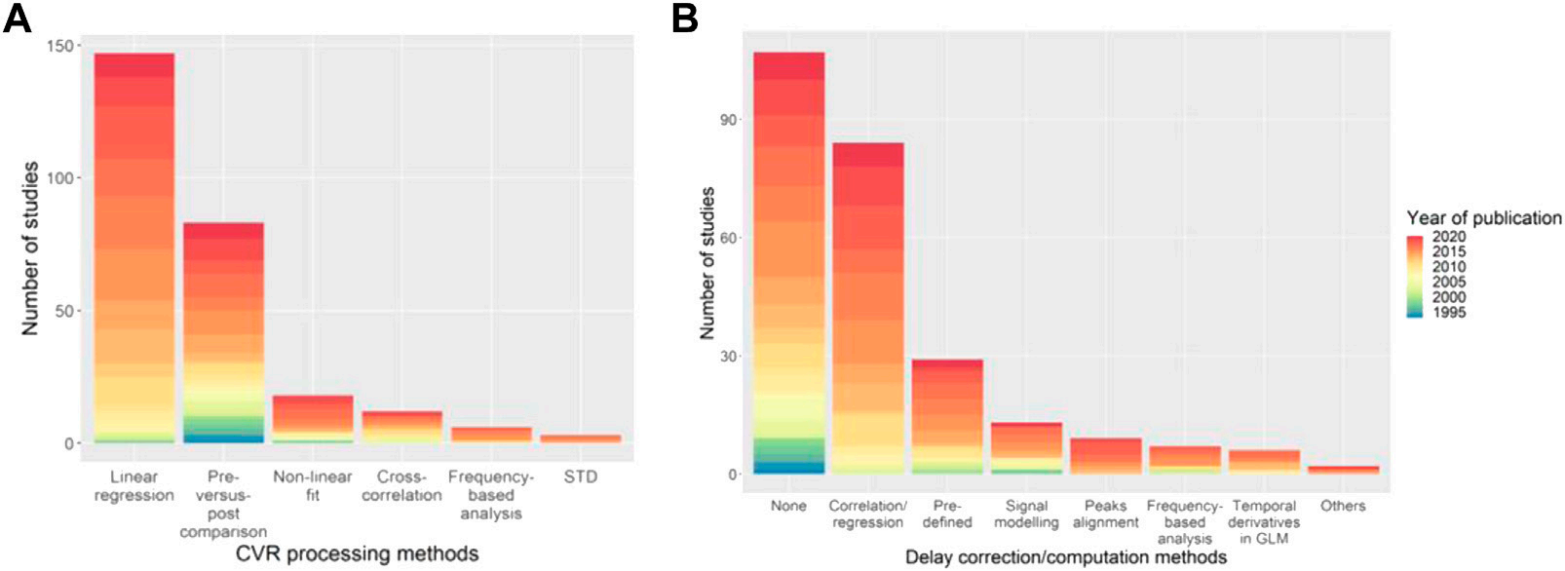
Distribution of the **(A)** CVR processing and **(B)** delay computation methods with the associated year of publication of the paper. The category “Others” in **(B)** includes deconvolution to find the HRF between the EtCO_2_ and the MRI signal, and GLM with two (“fast” and “slow”) regressors. STD, standard deviation of MRI signal; HRF, haemodynamic response function; GLM, general linear model.

The authors apologize for this error and state that this does not change the scientific conclusions of the article in any way. The original article has been updated.

