# Supplementary material for: Cerebrovascular Reactivity Measurement Using Magnetic Resonance Imaging: A Systematic Review
Source: Front Physiol. 2021 Feb 25;12:643468. doi: 10.3389/fphys.2021.643468 (PMC7947694; doi:10.3389/fphys.2021.643468)
Supplement: Supplementary file 1 [file Table_1.DOCX]

Supplementary Material

# Full search strategy

1. magnetic resonance/ or MR.mp. [mp=ti, ab, ot, nm, hw, fx, kf, ox, px, rx, ui, sy, tn, dm, mf, dv, kw, dq]
2. Blood*oxygen*level*dependent/ or BOLD.mp. [mp=ti, ab, ot, nm, hw, fx, kf, ox, px, rx, ui, sy, tn, dm, mf, dv, kw, dq]
3. susceptibility weight*/ or T2.mp. [mp=ti, ab, ot, nm, hw, fx, kf, ox, px, rx, ui, sy, tn, dm, mf, dv, kw, dq]
4. diffusion imaging/ or perfusion imaging.mp. [mp=ti, ab, ot, nm, hw, fx, kf, ox, px, rx, ui, sy, tn, dm, mf, dv, kw, dq]
5. ASL/ or arterial spin labelling.mp. [mp=ti, ab, ot, nm, hw, fx, kf, ox, px, rx, ui, sy, tn, dm, mf, dv, kw, dq]
6. phase-contrast MRI.mp. [mp=ti, ab, ot, nm, hw, fx, kf, ox, px, rx, ui, sy, tn, dm, mf, dv, kw, dq]
7. dual-echo imaging/ or dual-echo MRI.mp. [mp=ti, ab, ot, nm, hw, fx, kf, ox, px, rx, ui, sy, tn, dm, mf, dv, kw, dq]
8. functional magnetic resonance/ or fMRI.mp. [mp=ti, ab, ot, nm, hw, fx, kf, ox, px, rx, ui, sy, tn, dm, mf, dv, kw, dq]
9. 1 or 2 or 3 or 4 or 5 or 6 or 7 or 8
10. cerebrovascular reactivity/ or CVR.mp. [mp=ti, ab, ot, nm, hw, fx, kf, ox, px, rx, ui, sy, tn, dm, mf, dv, kw, dq]
11. cerebrovascular capacity/ or CVC.mp. [mp=ti, ab, ot, nm, hw, fx, kf, ox, px, rx, ui, sy, tn, dm, mf, dv, kw, dq]
12. vasodilation/ or vasoconstriction.mp. [mp=ti, ab, ot, nm, hw, fx, kf, ox, px, rx, ui, sy, tn, dm, mf, dv, kw, dq]
13. vascular resistance/ or cerebrovascular reserve.mp. [mp=ti, ab, ot, nm, hw, fx, kf, ox, px, rx, ui, sy, tn, dm, mf, dv, kw, dq]
14. 10 or 11 or 12 or 13
15. carbon dioxide/ or CO2/ or carbogen.mp. [mp=ti, ab, ot, nm, hw, fx, kf, ox, px, rx, ui, sy, tn, dm, mf, dv, kw, dq]
16. hypercapnia/ or hypercapnic.mp. [mp=ti, ab, ot, nm, hw, fx, kf, ox, px, rx, ui, sy, tn, dm, mf, dv, kw, dq]
17. hyperoxia/ or hypoxia.mp. [mp=ti, ab, ot, nm, hw, fx, kf, ox, px, rx, ui, sy, tn, dm, mf, dv, kw, dq]
18. breath-hold*/ or breath hold*/ or BH.mp. [mp=ti, ab, ot, nm, hw, fx, kf, ox, px, rx, ui, sy, tn, dm, mf, dv, kw, dq]
19. hyperventilation.mp. [mp=ti, ab, ot, nm, hw, fx, kf, ox, px, rx, ui, sy, tn, dm, mf, dv, kw, dq]
20. respiract.mp. [mp=ti, ab, ot, nm, hw, fx, kf, ox, px, rx, ui, sy, tn, dm, mf, dv, kw, dq]
21. end-tidal CO2/ or end-tidal carbon dioxide.mp. [mp=ti, ab, ot, nm, hw, fx, kf, ox, px, rx, ui, sy, tn, dm, mf, dv, kw, dq]
22. end-tidal O2/ or end-tidal oxygen.mp. [mp=ti, ab, ot, nm, hw, fx, kf, ox, px, rx, ui, sy, tn, dm, mf, dv, kw, dq]
23. resting state/ or resting-state.mp. [mp=ti, ab, ot, nm, hw, fx, kf, ox, px, rx, ui, sy, tn, dm, mf, dv, kw, dq]
24. acetazolamide.mp
25. 15 or 16 or 17 or 18 or 19 or 20 or 21 or 22 or 23 or 24
26. 9 and 14 and 25
27. limit 26 to human
28. remove duplicates from 27
